# CB2R activation enhances tumor-associated macrophages-mediated phagocytosis of glioma cell

**DOI:** 10.1016/j.heliyon.2024.e40806

**Published:** 2024-11-28

**Authors:** Siyuan Lu, Xuezhu Chen, Yang Yang, Junlong Li

**Affiliations:** a Office of Scientific Research Administration, The First Affiliated Hospital of Chongqing Medical University, Chongqing, 400016, China; b Department of Radiology, Affiliated People's Hospital of Jiangsu University, Zhenjiang, 212002, Jiangsu, China; c Department of Pathology, Public Health Medical Center, Chongqing, 400036, China; d Institute of Pathology and Southwest Cancer Center, Southwest Hospital, Third Military Medical University (Amy Medical University), Chongqing, 400038, China; e Office of Scientific Research Administration, Southwest Hospital, Third Military Medical University (Army Medical University), Chongqing, 400038, China

**Keywords:** Cannabinoid receptor 2, JWH133, Tumor-associated macrophages, Phagocytosis, CD36

## Abstract

**Background:**

Cannabinoid administration has demonstrated promising anti-tumor effects for glioblastoma (GBM) by inhibiting glioma cell proliferation and inducing glioma cell death. However, the impact of cannabinoids and endocannabinoid receptors on immune cells within the tumor microenvironment (TME) remains largely unexplored. Tumor-associated macrophages (TAMs), the most abundant immune cells in the TME, and their mediated phagocytosis of tumor cells have shown potential in preclinical xenografts of various human malignancies. This study aimed to investigate the effect and mechanism of endocannabinoid receptor 2 (CB2R) on TAMs-mediated phagocytosis in xenografted mice with GL261-GFP cell lines.

**Methods:**

We measured the phagocytic activity using immunofluorescence and flow cytometry, and we used the IVIS Spectrum System for bioluminescent imaging to track the growth of the tumor.

**Results:**

Our findings demonstrated that administering JWH133, a selective CB2R agonist, significantly boosted TAMs-mediated phagocytosis. However, administering AM630, a selective CB2R antagonist, significantly inhibited TAMs-mediated phagocytosis. Mechanistically, CB2R activation upregulated the expression of CD36 on TAMs, a scavenger receptor known to facilitate phagocytosis. Furthermore, sulfo-N-succinimidyl oleate (SSO), an irreversible CD36 inhibitor, could reverse the CB2R activation-induced enhancement of phagocytosis by TAMs. Additionally. JHW133 also effectively augmented the chemotherapeutic efficacy of temozolomide.

**Conclusion:**

Overall, our findings show that CB2R activation promotes TAMs-mediated phagocytosis of tumor cells by enhancing CD36 expression, implying that JWH133 could be a useful therapeutic approach to improving chemotherapeutic efficacy against GBM.

## 1 Introduction

Glioblastoma (GBM) is the most common and lethal brain tumor [1]. Despite various therapeutic approaches involving maximal safe surgical resection and chemoradiotherapy, over 95 % of GBM patients succumb within 5 years of diagnosis [2]. With the development of several novel treatments and approaches over the last decade, glioma patients' survival rates have not improved significantly. Extensive evidence suggests that the tumor microenvironment (TME) significantly contributes to the malignant growth and progression of GBM [[3], [4], [5]]. Extracellular matrix, soluble substances, blood vessels, parenchymal cells, and infiltrating immune cells are some of the elements that make up this microenvironment. Among these components, tumor-associated macrophages (TAMs) emerge as key players within GBM. What’s more, brain-resident microglia and peripheral monocyte-derived macrophages collectively termed TAMs in the tumor microenvironment [6]. TAMs are notably abundant in GBM tumors and exert a significant influence on tumor growth [7]. Their infiltration has been correlated with glioma progression and tumor grade, and it serves as a predictor of poor survival outcomes for GBM patients [8,9]. Understanding the intricate interactions between TAMs and the GBM microenvironment is crucial for devising effective therapeutic strategies against this aggressive form of GBM. Recent studies found that TAMs can engulf tumor cells and deliver tumor-specific antigens, inducing adaptive anti-tumor immunity [10,11]. Therefore, there is increasing interest in using cancer immunotherapy to target phagocytic activity of TAMs.

Cannabinoids, which are the bioactive constituents present in *Cannabis sativa* and its derivatives, have garnered widespread recognition for their diverse therapeutic applications against ailments including cardiovascular disorders, asthma, glaucoma, and tumors [12]. Cannabinoid receptor-1 (CB1R) and CB2R are the two endocannabinoid receptors that have been thoroughly investigated and cloned in vitro thus far [13]. Central nervous system presynaptic nerve terminal membranes are the predominant location of CB1R, which modulates cannabinoid psycho-activity and regulates neurotransmitter release [14]. Conversely, CB2R, primarily abundant in glial cells and is involved in immune regulation, notably documented in the treatment of neurological disorders like neurodegenerative diseases [15], multiple sclerosis [16], stroke [17], and spinal cord injury [18] by regulating microglial activity. Numerous tumor forms have been reported to have CB2R overexpression in their cells, making them attractive targets for cancer treatment [19]. Previous research has indicated a direct correlation between CB2R expression levels and glioma malignancy [12]. Increasing evidence suggests the potent anti-tumor properties of cannabinoids, which can be effectively utilized in the treatment of GBM. The therapeutic efficacy of cannabinoids relies on inducing tumor cell death by inhibiting tumor proliferation and angiogenesis, consequently impeding tumor growth [20]. Cannabinoids have been found to suppress the invasiveness and stem-like characteristics of GBM tumors [21]. After receiving cannabinoid therapy, patients with GBM have shown encouraging survival outcomes in recent phase II clinical trials [22]. Interestingly, localized administration of the selective CB2R agonist JWH133 caused glioma cell death [23] and glioma cell generation of vascular endothelial growth factor (VEGF) reduction [24], leading to significant glioma regression. Traditionally, CB2R receptors were thought to be peripheral cannabinoid receptors, primarily localized in peripheral tissues and immune cells, where they regulated inflammation and immune responses [13]. However, the impact of cannabinoids and CB2R activation on TAMs-mediated phagocytosis in glioma remains unknown.

This study aimed to examine the mechanism and effect of CB2R activation on TAMs-mediated phagocytosis using the GL261 glioma cell line. Furthermore, we evaluated the therapeutic potential of combining JWH133 and temozolomide (TMZ).

## 2 Materials and methods

### 2.1 Animals

Male C57BL/6 mice, aged 4–6 weeks, were obtained from the Experimental Animal Centre at the Third Military Medical University and were maintained in the Laboratory Animal Centre of Southwest Hospital (Gaotanyan Street No.30, Shapingba District, Chongqing, China). In vivo experiments did not involve randomization. The mice were housed in a temperature-controlled environment devoid of particular pathogens, with a standard 12-h light/dark cycle and unrestricted access to food and water. The experimental protocols follow the Animal Research: Reporting in Vivo Experiments criteria. The Ethics Committee of the Third Military Medical University approved the experimental protocols (AMUWEC20222930), which were conducted following the Guide for the Care and Use of Laboratory Animals.

### 2.2 Intracranial GBM xenografts and treatment

Professor Haofei Liu (Third Military Medical University, Chongqing, China) generously donated GL261 cells, a murine GBM cell line generated from C57BL/6 mice [25]. The GL261 cells originated from the National Cancer Institute in Frederick, Maryland [26]. To maintain cellular proliferation at 37 °C, 10 % fetal bovine serum (FBS, Gibco), was added to DMEM/F12 (HyClone). GL261 cells were transfected with pLV-GFP-luciferase (Luc) lentivirus, as previously described; flow cytometry was used to select GFP-positive GL261-GFP-Luc cells to generate a Luc-stable cell line [25]. Ketamine (50 mg/kg) and xylazine (5 mg/kg) were administered intraperitoneally into C57BL/6 to induce anesthesia in the GL261 tumor model. Following shaving, mouse heads were positioned within a stereotaxic frame. Following alcohol and betadine sterilization of the scalp, a midline scalp incision measuring 1 cm was performed to gain access to the cranium. Following that, the periosteum was extracted using 4 % H_2_O_2_. Next, starting from the bregma, a burr incision was performed 2 mm laterally and 0.8 mm anteriorly to the midline. A 10 μL syringe bearing a 33 G needle was withdrawn 0.5 mm from a depth of 3 mm to create a reservoir. Using a micro infusion pump, 10^4^ tumor cells in a volume of 3 μL were administered at a rate of 0.5 μL/min. After being in place for 5 min, the syringe was removed. Bone wax was used to fill the burr hole, and the skin was cleaned and conglutinated. At least 6 mice in each group were housed in a warm cage until they recovered completely.

On the seventh day following tumor implantation, mice were assigned to either the control or treatment group according to the bioluminescent signal of the initial tumor burden. To determine the effect of CB2R manipulation on tumor growth with or without TMZ treatment, mice were treated with vehicle, TMZ (5 mg/kg, i.p., Selleckchem, S1237), AM630 (1.0 mg/kg, Tocris Bioscience, 1120), JWH133 (1.0 mg/kg, Tocris Bioscience, 1343), or the combination of TMZ and JWH133. JWH133 and AM630 were intraperitoneally injected every day from day 3 after tumor cell implantation for 10 days. Five TMZ intraperitoneal injections were administered every 2 days beginning on the third day following tumor cell implantation [26]. To further verify whether CB2R enhances phagocytosis by up-regulating CD36 on tumor-associated macrophages, the xenografted mice were intraperitoneally injected with the sulfo-N-succinimidyl oleate (SSO, 15 mg/kg, MedChemExpress, HY-112847A), an irreversible inhibitor of the CD36 [27,28], every day from day 3 after tumor cell implantation for 10 days. Six mice per treatment group were randomly selected for live imaging, immunofluorescence, flow cytometry, and western blot. There are different batches of samples for above experiments and examined at day 14 after implantation. The sample size was at least nine mice per group for animal survival between groups.

### 2.3 Immunofluorescence

The protocols for immunofluorescence procedures followed those outlined by Yang et al. [29]. Briefly, the mice in each group were deeply anesthetized and perfused transcardially with 0.01M PBS followed by 4 % paraformaldehyde in 0.01M PBS. The whole brains were isolated, post-fixed in 4 % paraformaldehyde for 24–48 h, and then stored in 30 % sucrose in 0.01M PBS solution for 48 h for cryoprotection. After embedding and freezing with optimal cutting temperature compound (OCT), brains were sectioned into 30 μm-thick using a cryostat (Leica). The cryostat brain slices were treated with primary antibodies in 1 % bovine serum albumin (BSA), then the slices were incubated with mouse anti-CD11b (ab133357, 1:500, Abcam, Cambridge, MA) and chicken anti-GFP (ab13970, 1:1,000, Abcam) and maintained at 4 °C for the night. Following incubation, sections were washed with 0.01 M phosphate-buffered saline and subsequently labeled with Alexa Fluor Cy3 and 488 conjugated secondary antibodies (Jackson ImmunoResearch, West Grove, PA) at a dilution of 1:500 for 2 h at room temperature (RT). After that, sections were embedded in Vectashield mounting solution (Vector Laboratories, Burlingame, CA) and counterstained with 4′,6-diamidino-2-phenylindole (Santa Cruz Biotechnology, Santa Cruz, CA) before being photographed using a Zeiss confocal microscope (LSM780; Zeiss, Thornwood, NY). For quantification, the phagocytosis cell percentage was determined in five randomly chosen fields for each of the at least 6 mice per group.

### 2.4 Flow cytometry analyses

In the flow cytometry experiment, the mice in each group underwent deep anesthesia and transcardial perfusion with 0.01M PBS. The whole brains were dissected under a stereo fluorescence microscope (Nikon) to isolate the GFP-positive tissues with a sterile scalpel. The collected GFP-positive tissues were dissected into small pieces using scissors. These pieces were then placed in C-Tubes (Miltenyi Biotec, 130-096-334) containing a digestion cocktail (11088882001, Roche, Indianapolis, IN; MO D7291-2 MG, Sigma-Aldrich, St. Louis) containing 1 mg/mL collagenase D and 30 mg/mL DNase I (11088882001, Roche) in complete RPMI 1640 medium (10 % FBS). The temperature was maintained at 37 °C for 15 min. Following that, the tissue was gently dissociated utilizing a MACS and subsequently incubated at 37 °C for an additional 15 min. The homogenate was subsequently filtered through a 70 μm strainer and centrifuged at 4 °C for 10 min at 500×*g*. The resulting pellet was resuspended in RPMI-1640 medium supplemented with 10 % FBS. Following this, an immune cell enrichment step was performed using density gradient centrifugation with 30 % and 70 % Percoll (Fisher Scientific, 10607095) in RPMI 1640 medium containing 10 % FBS. Upon centrifugation for 20 min at 20 °C and 2000×*g* (without using any force during acceleration or deceleration), the immune cells-containing middle transparent layer was carefully extracted.

Singular-cell suspensions were generated from complete GL261-GFP-Luc allografts as previously documented [25] to quantify the proportion of TAMs exhibiting phagocytosis in the tumor tissue of the GBM model. To inhibit non-specific and Fc-mediated binding, the cells were incubated in ice with anti-CD16/CD32 (553141; BD Biosciences, San Jose, CA) antibodies. Following a 10-min incubation period, three washes and an additional 60 min of dark incubation were required for the GL261-GFP-Luc allograft single-cell suspensions to be treated with APC anti-mouse CD11b antibody (101211, 1:1000; Biolegend, San Diego, CA). Isotype controls were employed for every staining attempt. The data acquisition process was conducted on BDFACSAria II, and FlowJo X 10.0.7 (TreeStar, Ashland, OR) was utilized to analyze and present the data.

### 2.5 Living imaging

To measure tumor growth, tumor-bearing mice were anesthetized with isoflurane 14 days following tumor implantation and administered an intraperitoneal injection of D-Luciferin (150 mg/kg body weight; Cat# P1043; Promega, Madison, WI). After 10 min of being moved to the in vivo imaging system (IVIS) 100 imaging equipment (Caliper Life Sciences, Alameda, CA), the animals’ luminescence was detected. Following this, the data were analyzed utilizing the Living Image 2.5 software (Coronar Life Sciences).

### 2.6 Western blotting

The flow cytometry-sorted CD11b-positive TAMs of different groups were lysed in accordance with a process published in a prior work [6]. Specifically, ice-cold RIPA buffer (Sigma-Aldrich) was utilized, which had been enriched with a protease inhibitor cocktail (Roche). The levels of protein were quantitatively assessed through the Enhanced BCA Protein Assay Kit (Beyotime, Beijing, China). Following that, polyvinylidene fluoride membranes were utilized to transfer the separated samples, each of which contained 20 mg of protein, via 10 % SDS-PAGE (Roche). Primary antibodies against CD36 (rabbit, ab252923, 1:1000; Abcam) and GAPDH (mouse, ab8245, 1:1000; Abcam) were incubated at 4 °C overnight after the membranes had been incubated at RT for 2 h in Tris-buffered saline with 0.1 % Tween 20 detergent containing BSA and 0.05 % Tween 20. Following this, the membranes were incubated at RT for 1 h with an HRP-linked secondary antibody The protein bands were identified utilizing the WesternBright ECL kit (Advansta, Menlo Park, CA) in conjunction with the ChemiDocTM XRS C imaging system (Bio-Rad, Berkeley, CA; Densitometry was performed on each of the membranes utilizing the Image LabTM software (Bio-Rad).

### 2.7 Statistical analysis

Statistical analyses in this study were conducted using GraphPad Prism 8.0 and SPSS 18.0 software. The Kolmogorov-Smirnov test was utilized to evaluate whether the data were normally distributed. Comparisons between 2 groups were analyzed using 2-tailed Student *t* tests. Tukey's post hoc test was applied after one-way ANOVA (one factor) was utilized to examine intergroup comparisons. Survival rates were analyzed using the Kaplan-Meier (K-M) method, and the differences between groups were compared using the log-rank test. The format of mean ± SEM was used to express the data, and a P value < 0.05 was deemed to demonstrate statistical significance.

## 3 Results

### 3.1 CB2R activation impairs GBM growth and improves the survival of xenografted mice

**Fig. 1:**
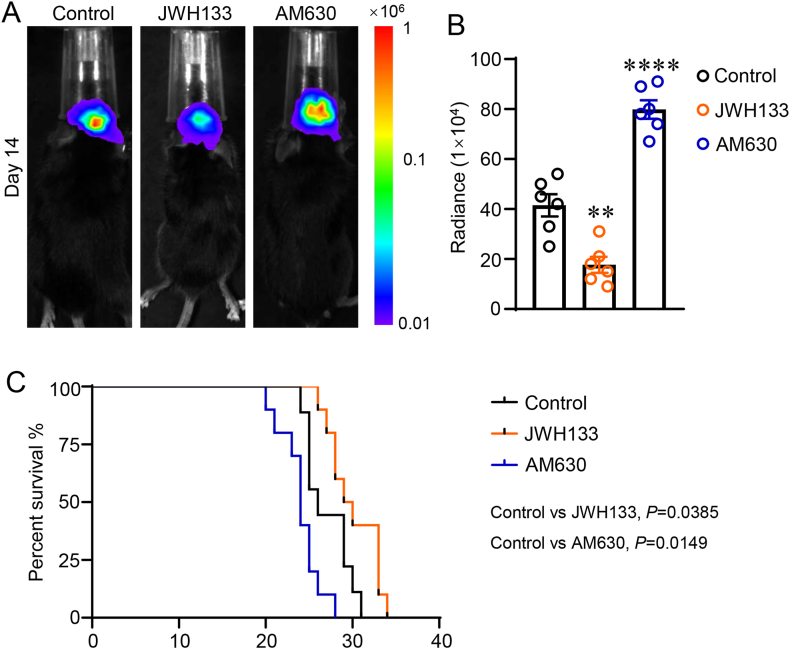
CB2R activation impairs GBM growth and improves the survival of xenografted mice. (A) Images of the bioluminescence intensity obtained on day 14 post-implantation of representative mice from each treatment group. (B) Tumor progression quantification for each mouse based on flux data obtained using bioluminescence intensity photometry. (C) Kaplan-Meier (K–M) analysis of the survival of each treatment group's mice carrying GL261 xenografts. The format of mean ± SEM is used to display the data. Each group contains n = 6–10 mice. One-way ANOVA with the Bonferroni correction was used to determine ∗*P* < 0.05 and ∗∗∗∗*P* < 0.0001 relative to the control group (B). The K-M method was used for survival analysis, and the log-rank test was used as a comparison (C).

We created GBM xenografts from Luc-expressing GL261 cells to determine whether CB2R activation affects the progression of GBM in vivo. Mice were treated with JWH133 (a selective CB2R agonist), AM630 (a selective CB2R antagonist), or the vehicle 3 days after GL261-Luc cells were implanted. On day 14 post-implantation, the tumor growth was tracked using an IVIS and a bioluminescence signal. In comparison to the Control group, JWH133 therapy inhibited GBM growth, according to bioluminescence analysis (Fig. 1A–B, *P* < 0.01). On the contrary, mice treated with AM630 experienced a considerably quicker progression of GBM in comparison to the Control group (Fig. 1A–B, *P* < 0.0001). The K-M survival analysis showed that the administration of JWH133 produced a survival advantage over the Control group in line with the reported consequences on tumor growth (Fig. 1C, *P =* 0.0385). In contrast, survival was significantly reduced in mice treated with AM630 relative to the Control group (Fig. 1C, *P =* 0.0149). Overall, these findings demonstrated that CB2R activation impedes GBM growth and enhances the survival of xenografted mice.

### 3.2 CB2R activation promotes TAMs-mediated phagocytosis of glioma cells

**Fig. 2:**
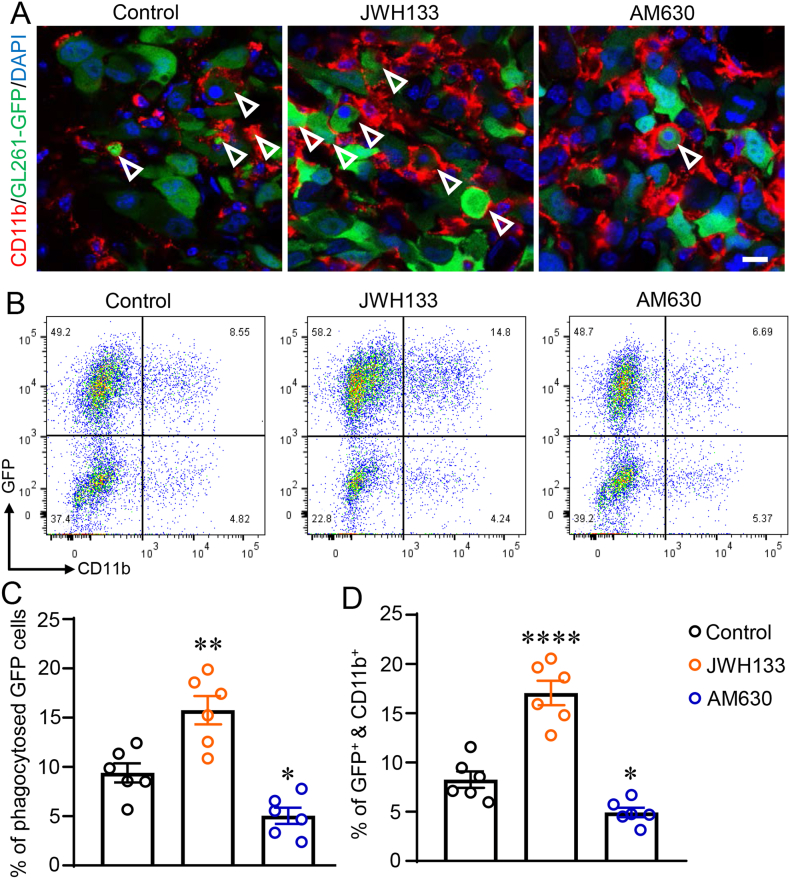
CB2R activation promotes TAMs-mediated phagocytosis of glioma cells. (A) Representative immunofluorescence images of the xenografted brain stained with GFP (green) and CD11b (red) in mice injected with Vehicle, JWH133 or AM630. White arrows indicated the phagocytes. Scale bar: 10 μm. (B) Representative flow cytometry plots depicting the phagocytosis of GFP-labeled glioma cells with CD11b-labeled TAMs in the presence of Vehicle, JWH133, or AM630. (C) The determination of the proportion of phagocytosed GFP cells in mice that received injections of Vehicle, JWH133, or AM630. (D) Quantification of the percentage of GFP and CD11b double-positive cells among all the sorted cells in mice injected with Vehicle, JWH133, or AM630. The format of mean ± SEM is used to display the data. Each group contains n = 6 mice. One-way ANOVA with the Bonferroni correction was used to determine ∗*P* < 0.05, ∗∗*P* < 0.01 and ∗∗∗∗*P* < 0.0001 relative to the control group. (For interpretation of the references to colour in this figure legend, the reader is referred to the Web version of this article.)

To assess whether CB2R activation enhances TAMs-mediated phagocytosis of glioma cells, we administered JWH133 and AM630 to xenografted mice bearing GL261-GFP cell line tumors for 10 days. Histologically, the CD11b, labeled with RFP, served as a marker for TAMs. Consequently, phagocytes were identified by the presence of red protrusions containing GFP-positive cells. This method facilitated the characterization of TAMs-mediated phagocytosis within the experimental model. Our findings demonstrated that treatment with JWH133 significantly increased the phagocytosis of GFP-positive glioma cells by TAMs in comparison to the Control group (Fig. 2 A and C, *P* *<* 0.01). Conversely, AM630 treatment led to a notable inhibition of TAMs-mediated phagocytosis compared to the Control group (Fig. 2 A and C, *P* < 0.05). Following the results of the flow-based phagocytosis assay, treatment with JWH133 significantly increased phagocytosis mediated by TAMs in comparison to the Control group (Fig. 2 B and D, *P* < 0.0001), whereas treatment with AM630 substantially decreased this process in comparison to the Control group (Fig. 2 B and D, *P* < 0.05). Thus, CB2R activation is a crucial mediator of the phagocytic activity of TAMs, according to the collective findings presented herein.

### 3.3 CB2R activation promotes TAMs-mediated phagocytosis of glioma cells via increasing the expression of CD36 in TAMs

**Fig. 3:**
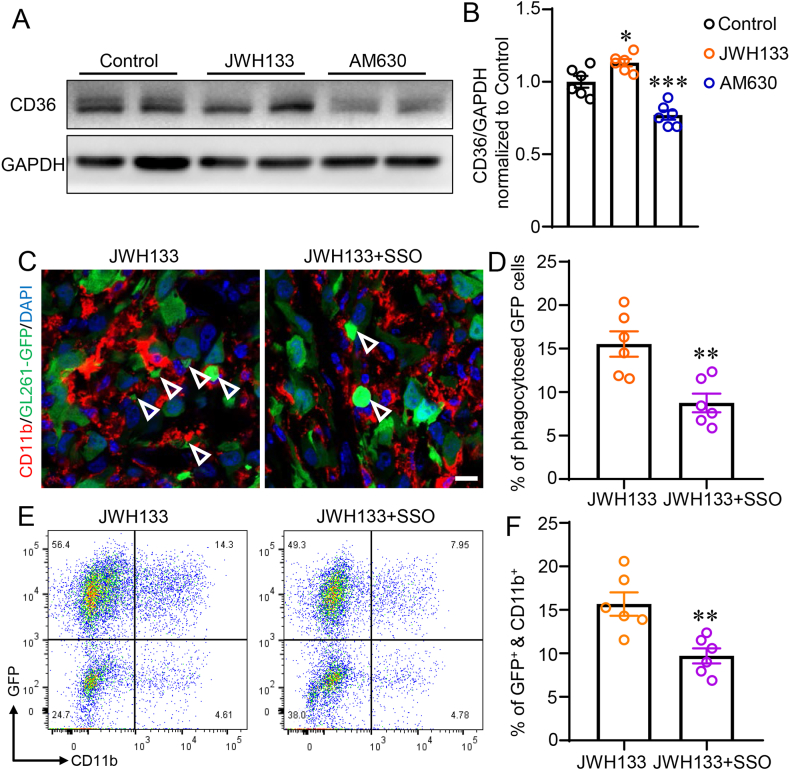
CB2R activation promotes TAMs-mediated phagocytosis of glioma cells via increasing the expression of CD36 in TAMs. (A) Representative western blot bands of CD36 and GAPDH from flow cytometry sorted CD11b positive cells in xenografted mice injected with Vehicle, JWH133, or AM630 at day 14 after implantation. (B) Quantification of CD36 and GAPDH from flow cytometry sorted CD11b positive cells with Vehicle, JWH133, or AM630 at day 14 after implantation. (C) Representative immunofluorescence images of xenografted brain tissue depict GFP (green) and CD11b (red) staining in mice injected in each group. White arrows indicated the phagocytes. Scale bar: 10 μm. (D) The determination of the proportion of phagocytosed GFP cells in mice in each group. (E) Representative flow cytometry plots depicting the phagocytosis of GFP-labeled glioma cells with CD11b-labeled TAMs in each group. (F) Quantification of the percentage of GFP and CD11b double-positive cells among all the sorted cells in each group. The format of mean ± SEM is used to display the data. Each group contains n = 6 mice. One-way ANOVA with the Bonferroni correction was used to determine ∗*P* < 0.05 and ∗∗∗*P* < 0.001 relative to the control group (B). Student *t*-test was used to determine ∗∗*P* < 0.01 relative to the JWH133 group (D and F). (For interpretation of the references to colour in this figure legend, the reader is referred to the Web version of this article.)

CD36, which belongs to the class B scavenger receptor family, is essential for the phagocytic activity of macrophages, monocytes, and microglia, among other immune cells. Given its role in phagocytosis, CD36 has garnered significant interest in studies focused on inflammation, atherosclerosis, and neurodegenerative diseases, among other areas [30]. To investigate whether CD36 mediates the CB2R-induced phagocytic activity of TAMs in glioma, we sorted CD11b-positive TAMs using flow cytometry and examined the expression of CD36 in each group. According to Western blotting analysis findings, TAMs treated with JWH133 had considerably higher CD36 expression than the Control group (Fig. 3A–B, *P* < 0.05). However, AM630 treatment dramatically decreased CD36 expression in TAMs as compared to the Control group (Fig. 3A–B, *P* < 0.001). What’s more, to verify whether CB2R enhances phagocytosis by increasing CD36 levels on tumor-associated macrophages, we treated xenografted mice with SSO, an irreversible CD36 inhibitor [27]. Remarkably, immunofluorescence and flow cytometry results demonstrate that SSO treatment reversed the CB2R activation-induced enhancement of phagocytosis by TAMs (Fig. 3C–F, *P* < 0.01). These findings suggest that CB2R activation increases CD36 expression, which in turn promotes the phagocytic activity of TAMs. This sheds light on a potential mechanism underlying the observed effects of CB2R modulation on TAMs-mediated phagocytosis in glioma.

### 3.4 JWH133 enhances the therapeutic efficacy of TMZ

**Fig. 4:**
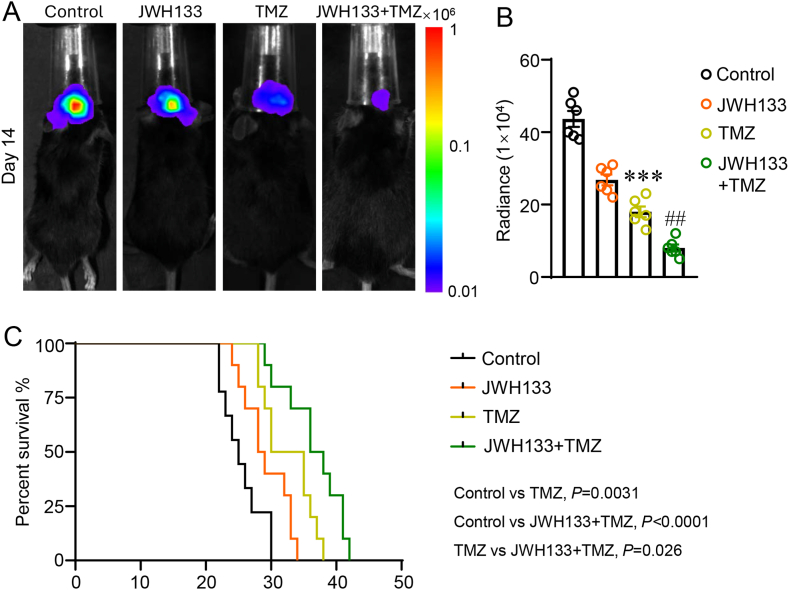
JWH133 enhances the therapeutic efficacy of temozolomide (TMZ). (A) Images of the bioluminescence intensity obtained on day 14 post-implantation of representative mice from each treatment group. (B) Tumor progression quantification for each mouse based on flux data obtained using bioluminescence intensity photometry. (C) Kaplan-Meier (K–M) analysis of the survival of each treatment group's mice carrying GL261 xenografts. Each group contains n = 6–10 mice. One-way ANOVA with the Bonferroni correction was used to determine ∗∗∗*P* < 0.001 relative to the control group and ^##^*P* < 0.01 relative to the TMZ group (B). The K-M method was used for survival analysis, and the log-rank test was used as a comparison (C).

Due to resistance in certain patients, TMZ, a primary chemotherapy for GBM that is well-known for its capacity to cross the blood-tumor barrier and cause cytotoxicity by breaking DNA double strands, is not always effective. This resistance is often correlated with mutations in isocitrate dehydrogenase 1 and elevated levels of O6-methyl guanine-DNA methyltransferase promoter methylation [31]. A synergistic approach, combining TMZ with immunotherapeutic strategies, emerges as a promising avenue of investigation. In this part, we evaluated whether JWH133 enhances the chemotherapeutic efficacy of TMZ. Mice implanted with GL261-Luc cells were administered either JWH133 with or without concurrent TMZ therapy or a control vehicle. Tumor progression was assessed 14 days post-implantation through bioluminescence imaging using the IVIS. When comparing the TMZ treatment group to the control group, the study showed a significant decrease in GBM growth (Fig. 4A–B, *P* < 0.001). Interestingly, JWH133 with TMZ therapy significantly increased the anti-tumoral efficacy of TMZ alone (Fig. 4A–B, *P* < 0.01). The K-M survival analysis corroborated this inhibitory effect on tumor growth, showing that TMZ treatment alone was linked to a survival advantage over the control (Fig. 4C, *P =* 0.0031). Significantly, the co-treatment extended survival further than the TMZ treatment alone (Fig. 4C, *P =* 0.026). Collectively, these findings illuminate the potential of JWH133 to potentiate the therapeutic effectiveness of TMZ in combating GBM.

## 4 Discussion

TAMs are critical components of tumor immunity and are among the TME's most prevalent non-malignant cells. What’s more, TAMs are important innate immune cells that possess the ability to engulf tumor cells and present antigens specific to the tumor, which triggers adaptive immunity against the tumor [11]. Thus, modulating the phagocytic activity of TAMs for cancer immunotherapy is becoming an increasingly popular strategy. On the contrary, living tumor cells have developed strategies to elude phagocytosis by TAMs through the extensive expression of anti-phagocytic molecules such as CD47 [32,33]. Recent research suggests that the immunosuppressive phenotype is enhanced when glioma cells are phagocytosed [34]. Therefore, enhancing the phagocytic activity of TAMs presents a promising strategy for advancing cancer immunotherapy. Cannabinoids, the primary active compounds found in medical cannabis, exert a wide array of pharmacological effects across various diseases [12]. Numerous studies have highlighted their anti-proliferative and anti-invasive properties in different cancer types, including the inhibition of invasiveness and stem-like characteristics in GBM tumors [21]. Recent phase II clinical trials have shown promising survival outcomes in GBM patients following cannabinoid therapy [22]. Interestingly, glioma cell death and the inhibition of VEGF production in glioma cells have been caused by localized delivery of the selective CB2R agonist JWH133, and this has led to considerable glioma regression [24]. Although CB2R was previously believed to be exclusively expressed in immune cells, the impact of CB2R activation on TAMs-mediated phagocytosis in glioma remains poorly understood. To explore this issue, we selected the selective CB2R agonist, JWH133, to treat the xenografted mice in this study. Our results indicated that JWH133 treatment significantly enhanced TAMs-mediated phagocytosis in glioma.

While cannabinoids are known to activate CB2R, their use as CB2R receptor activators is limited. Firstly, cannabinoids often activate both CB1R and CB2R non-selectively. CB1R is predominantly located in the central nervous system and is responsible for the psychoactive effects of cannabinoids [14]. The activation of CB1R can lead to unwanted side effects such as altered mental state, dependence, and other neurological issues, which are undesirable in many therapeutic contexts [14]. Secondly, the legal status of cannabinoids varies greatly worldwide, which poses challenges for their development and clinical use. Thirdly, advances in medicinal chemistry have led to the development of selective CB2R agonists that do not affect CB1R. These selective agonists can provide the therapeutic benefits of CB2R activation without the drawbacks associated with cannabinoids. Therefore, in our study, JWH133 was chosen as a selective CB2R agonist for the treatment of xenografted mice, underscoring its suitability for therapeutic application.

One of the TME's most prevalent non-malignant cells, TAMs are essential for mediating tumor immunity. Macrophages/microglia can be categorized into two subpopulations: those that are classically activated (M1) and possess pro-inflammatory attributes; and those that are differentially activated (M2) and exhibit anti-inflammatory characteristics [35]. According to recent research, TAMs can be functionally divided into two groups: M1-type, which repress tumors, and M2-type, which support tumors. M2 TAMs are typically immuno-suppressive and allow GBM malignant tendencies to promote tumor growth, whereas M1 TAMs have an immune surveillance function [36]. According to research findings, CB2R activation may be able to reduce inflammation in stroke, traumatic brain injury, and neurodegenerative diseases by promoting anti-inflammatory M2 polarization and decreasing pro-inflammatory M1 polarization [17]. This suggests that CB2R activation facilitates the growth of GBM by promoting the polarization of M2 TAMs. A recent study discovered that the immune-checkpoint proteins CD276, programmed cell death ligand (PD-L) 1, and PD-L2 were expressed by the TAMs that engulfed the glioma cells and that this led the TAMs to become M2-like that drove immunosuppression by inhibiting the growth of activated T cells [34]. Another research also demonstrated that simvastatin promoted erythrocyte phagocytosis of macrophages/microglia via upregulating CD36 expression and facilitating M2 phenotype polarization [17]. However, phagocytes more likely present M1-like morphology [37,38]. The study directly explores the phagocytic role of TAMs induced by CB2R activation, rather than focusing solely on how CB2R regulates TAMs polarization into M1/M2 phenotypes [39]. This approach acknowledges the potential independent regulation of TAMs phagocytosis irrespective of their polarization states. Such methodology aids in a better understanding of how CB2R activation modulates phagocytic function of TAMs while circumventing oversimplification of TAMs into M1 and M2 classifications. Interestingly, our results found that CB2R activation enhanced TAMs-mediated phagocytosis in glioma, impaired GBM growth, and improved the survival of xenografted mice. Moreover, activating CB2R also improved the efficacy of TMZ in inhibiting GBM growth and increasing the lifespan of xenografted mice. Similarly, recent study also found that administration of submaximal doses of Δ(9)-Tetrahydrocannabinol and cannabidiol remarkably reduces the growth of glioma xenografts in both TMZ-sensitive and TMZ-resistant GBM [40].

CD36 is a class B scavenger receptor that has been shown to help transport a variety of substances into cells, including collagen, oxidized low-density lipoproteins, and fatty acids [30]. Consequently, CD36 plays a pivotal role in the phagocytic response of diverse immune cells, such as microglia, macrophages, and monocytes. A previous study indicated that CD36 is essential for the phagocytosis of apoptotic cells by macrophages [41]. Furthermore, in Alzheimer's disease, triggering receptor expressed on myeloid cells 2-induced overexpression of CD36 improved microglia phagocytosis of Aβ [42]. Our current work extends previous findings that upregulated the expression of CD36 enhanced the phagocytic activity of TAMs that more GFP glioma cells were engulfed, after CB2R activation. Therefore, CB2R activation induced CD36 upregulation mediated the TAMs-mediated phagocytosis in glioma. However, previous research has established CD36 as a pivotal biomarker for cancer, showing a negative association with patients’ outcomes [43]. Elevated levels of CD36 in glioma stem cells have been linked to enhanced proliferative abilities and faster tumor progression within living organisms. Targeting CD36, using either drug-based treatments or genetic modification techniques, resulted in diminished stem-like traits in these cells and a notable slowing of tumor expansion [43,44]. Furthermore, CD36 plays a role in hindering angiogenesis in GBM through its interaction with vasculostatin, a split product from the G protein-coupled receptor known as brain angiogenesis inhibitor I, which leads to endothelial cell apoptosis [45,46]. The specific effects of CD36 can vary greatly depending on the cell type and the ligands involved. The potential impact of CB2R activation on CD36 expression within glioma and endothelial cells is yet to be identified, which needs further investigation.

Our study has several limitations. First, while both brain-resident microglia and peripheral monocyte-derived macrophages are collectively named as TAMs and identified by CD11b, a more detailed classification would be beneficial, particularly for distinguishing the different function between macrophages and microglia in future research. Second, the mechanisms through which CB2R activation induces CD36 upregulation require further investigation. Third, since CD36 is also expressed in glioma stem cells and endothelial cells, specifically targeting CD36 in TAMs would provide stronger evidence for our findings.

## 5 Conclusion

Overall, our study elucidates that CB2R activation can promote TAMs-mediated phagocytosis of tumor cells by increasing CD36 expression, suggesting JWH133 could serve as a viable therapeutic approach to enhance the efficacy of chemotherapy in treating GBM.

## CRediT authorship contribution statement

**Siyuan Lu:** Methodology, Investigation, Data curation. **Xuezhu Chen:** Validation, Software, Methodology, Investigation, Formal analysis, Data curation. **Yang Yang:** Writing – review & editing, Writing – original draft, Validation, Funding acquisition, Conceptualization. **Junlong Li:** Writing – review & editing, Writing – original draft, Funding acquisition, Data curation, Conceptualization.

## Ethics statement

The Ethics Committee of the Third Military Medical University approved the experimental protocols (AMUWEC20222930), which were conducted following the Guide for the Care and Use of Laboratory Animals.

## Data availability statement

Data will be made available on request.

## Funding declaration

This work was supported by the 10.13039/501100005230Natural Science Foundation of Chongqing (Grant No. CSTB2022NSCQ-MSX1179), the Postdoctoral Innovative Talent Support Program of Chongqing (Grant No. CQBX2021008), and the 10.13039/501100002858China Postdoctoral Science Foundation (Grant No. 2022M723868).

## Declaration of competing interest

The authors declare that they have no known competing financial interests or personal relationships that could have appeared to influence the work reported in this paper.

## References

[1] Horbinski C. (2022). Clinical implications of the 2021 edition of the WHO classification of central nervous system tumours. Nat. Rev. Neurol..

[2] Wu W. (2021). Glioblastoma multiforme (GBM): an overview of current therapies and mechanisms of resistance. Pharmacol. Res..

[3] de Visser K.E., Joyce J.A. (2023). The evolving tumor microenvironment: from cancer initiation to metastatic outgrowth. Cancer Cell.

[4] Fang J. (2023). Exploring the crosstalk between endothelial cells, immune cells, and immune checkpoints in the tumor microenvironment: new insights and therapeutic implications. Cell Death Dis..

[5] Liu X. (2019). The reciprocal regulation between host tissue and immune cells in pancreatic ductal adenocarcinoma: new insights and therapeutic implications. Mol. Cancer.

[6] Shi Y. (2017). Tumour-associated macrophages secrete pleiotrophin to promote PTPRZ1 signalling in glioblastoma stem cells for tumour growth. Nat. Commun..

[7] Hambardzumyan D., Gutmann D.H., Kettenmann H. (2016). The role of microglia and macrophages in glioma maintenance and progression. Nat. Neurosci..

[8] Cassetta L., Pollard J.W. (2023). A timeline of tumour-associated macrophage biology. Nat. Rev. Cancer.

[9] Basheer A.S. (2021). Role of inflammatory mediators, macrophages, and neutrophils in glioma maintenance and progression: mechanistic understanding and potential therapeutic applications. Cancers.

[10] Zhou X., Liu X., Huang L. (2021). Macrophage-mediated tumor cell phagocytosis: opportunity for nanomedicine intervention. Adv. Funct. Mater..

[11] von Roemeling C.A. (2020). Therapeutic modulation of phagocytosis in glioblastoma can activate both innate and adaptive antitumour immunity. Nat. Commun..

[12] Pattnaik F. (2022). Cannabis: chemistry, extraction and therapeutic applications. Chemosphere.

[13] Callen L. (2012). Cannabinoid receptors CB1 and CB2 form functional heteromers in brain. J. Biol. Chem..

[14] Rodriguez J.J., Mackie K., Pickel V.M. (2001). Ultrastructural localization of the CB1 cannabinoid receptor in mu-opioid receptor patches of the rat Caudate putamen nucleus. J. Neurosci..

[15] Cassano T. (2017). Cannabinoid receptor 2 signaling in neurodegenerative disorders: from pathogenesis to a promising therapeutic target. Front. Neurosci..

[16] Docagne F. (2008). Therapeutic potential of CB2 targeting in multiple sclerosis. Expert Opin. Ther. Targets.

[17] Tao Y. (2016). Cannabinoid receptor-2 stimulation suppresses neuroinflammation by regulating microglial M1/M2 polarization through the cAMP/PKA pathway in an experimental GMH rat model. Brain Behav. Immun..

[18] Brownjohn P.W., Ashton J.C. (2012). Spinal cannabinoid CB2 receptors as a target for neuropathic pain: an investigation using chronic constriction injury. Neuroscience.

[19] Moreno E. (2019). The endocannabinoid system as a target in cancer diseases: are we there yet?. Front. Pharmacol..

[20] Hinz B., Ramer R. (2022). Cannabinoids as anticancer drugs: current status of preclinical research. Br. J. Cancer.

[21] Pisanti S. (2017). Cannabidiol: state of the art and new challenges for therapeutic applications. Pharmacol. Ther..

[22] Schultz S., Beyer M. (2017). GW pharmaceuticals achieves positive results in phase 2 proof of concept study in glioma. http://ir.gwpharm.com/static-files/cde942fe-555c-4b2f-9cc9-f34d24c7ad27.

[23] Sanchez C. (2001). Inhibition of glioma growth in vivo by selective activation of the CB(2) cannabinoid receptor. Cancer Res..

[24] Blazquez C. (2004). Cannabinoids inhibit the vascular endothelial growth factor pathway in gliomas. Cancer Res..

[25] Liu H. (2023). Neutralizing IL-8 potentiates immune checkpoint blockade efficacy for glioma. Cancer Cell.

[26] Szatmari T. (2006). Detailed characterization of the mouse glioma 261 tumor model for experimental glioblastoma therapy. Cancer Sci..

[27] Zhu G.Q. (2023). CD36(+) cancer-associated fibroblasts provide immunosuppressive microenvironment for hepatocellular carcinoma via secretion of macrophage migration inhibitory factor. Cell Discov.

[28] Wei Y. (2024). Dietary long-chain fatty acids promote colitis by regulating palmitoylation of STAT3 through CD36-mediated endocytosis. Cell Death Dis..

[29] Yang Y. (2022). MEC17-induced α-tubulin acetylation restores mitochondrial transport function and alleviates axonal injury after intracerebral hemorrhage in mice. J. Neurochem..

[30] Chen Y. (2022). CD36, a signaling receptor and fatty acid transporter that regulates immune cell metabolism and fate. J. Exp. Med..

[31] Gong L. (2022). Characterization of EGFR-reprogrammable temozolomide-resistant cells in a model of glioblastoma. Cell Death Discov.

[32] Gholamin S. (2017). Disrupting the CD47-SIRPalpha anti-phagocytic axis by a humanized anti-CD47 antibody is an efficacious treatment for malignant pediatric brain tumors. Sci. Transl. Med..

[33] Theruvath J. (2022). Anti-GD2 synergizes with CD47 blockade to mediate tumor eradication. Nat Med.

[34] Wu M. (2023). Phagocytosis of glioma cells enhances the immunosuppressive phenotype of bone marrow-derived macrophages. Cancer Res..

[35] Xie D., He M., Hu X. (2019). Microglia/macrophage diversities in central nervous system physiology and pathology. CNS Neurosci. Ther..

[36] Boutilier A.J., Elsawa S.F. (2021). Macrophage polarization states in the tumor microenvironment. Int. J. Mol. Sci..

[37] Yang S.S. (2016). High morphologic plasticity of microglia/macrophages following experimental intracerebral hemorrhage in rats. Int. J. Mol. Sci..

[38] Xia Y. (2020). Engineering macrophages for cancer immunotherapy and drug delivery. Adv Mater.

[39] Wang W. (2024). Identification of hypoxic macrophages in glioblastoma with therapeutic potential for vasculature normalization, 2024. Cancer Cell.

[40] Torres S. (2011). A combined preclinical therapy of cannabinoids and temozolomide against glioma. Mol Cancer Ther.

[41] Fadok V.A. (1998). CD36 is required for phagocytosis of apoptotic cells by human macrophages that use either a phosphatidylserine receptor or the vitronectin receptor (alpha v beta 3). J. Immunol..

[42] Yamanaka M. (2012). PPARgamma/RXRalpha-induced and CD36-mediated microglial amyloid-beta phagocytosis results in cognitive improvement in amyloid precursor protein/presenilin 1 mice. J. Neurosci..

[43] Hale J.S. (2014). Cancer stem cell-specific scavenger receptor CD36 drives glioblastoma progression. Stem Cell..

[44] Silver D.J. (2021). Severe consequences of a high-lipid diet include hydrogen sulfide dysfunction and enhanced aggression in glioblastoma. J. Clin. Invest..

[45] Kaur B. (2009). Vasculostatin inhibits intracranial glioma growth and negatively regulates in vivo angiogenesis through a CD36-dependent mechanism. Cancer Res..

[46] Klenotic P.A. (2010). Histidine-rich glycoprotein modulates the anti-angiogenic effects of vasculostatin. Am. J. Pathol..

