# Supplementary figures and images for: CB2R activation enhances tumor-associated macrophages-mediated phagocytosis of glioma cell
Source: Heliyon. 2024 Nov 28;10(23):e40806. doi: 10.1016/j.heliyon.2024.e40806 (PMC11650289; doi:10.1016/j.heliyon.2024.e40806)

Original Images for Figure 3A

| CD36 | 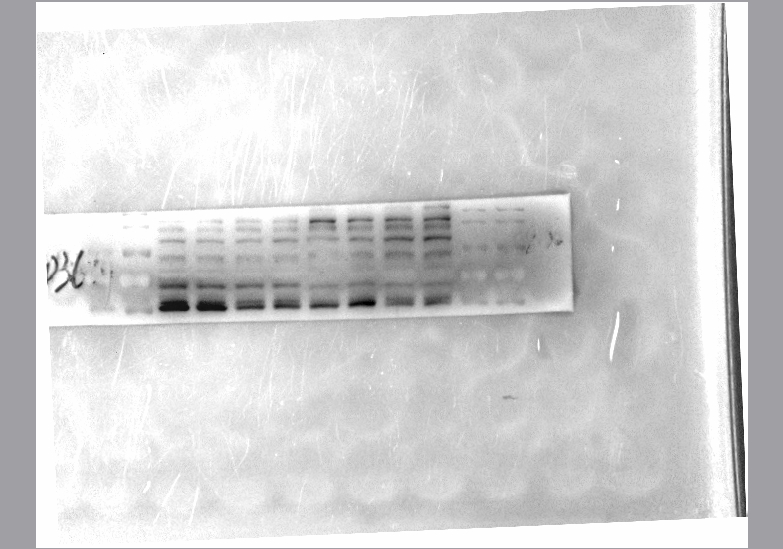 |
| --- | --- |
| GAPDH | 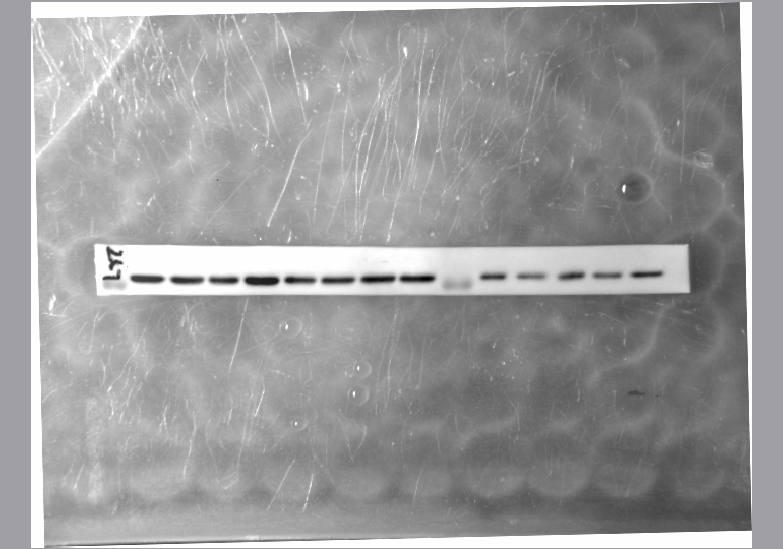 |

Supplement: Multimedia component 1 [file mmc1.docx]
